# Smart ROUV advances for enhanced navigation in the Suez Canal

**DOI:** 10.1038/s41598-025-23281-8

**Published:** 2025-11-07

**Authors:** Khaled Oqda, Kareem Mosaad Ibrahim, Ahmed Ibrahim Mustafa, Reem Gamal Allam, Muhamed Ahmed Saad, Ahmed Ibrahim Asl, Mohamed Al-Sayed Abdel Samie, Sara Abdel Fattah Ibrahim, Hassan Yahya Ghanem, Mohamed Mahmoud Habib, Mohamed Ali Metwalliy, Hanaa Salem Marie

**Affiliations:** 1 Mechatronics Department, Faculty of Engineering, Mansoura University, Mansoura, Egypt; 2 Faculty of Engineering, Delta University for Science and Technology, Gamasa, 35712 Egypt; 3 Faculty of Artificial Intelligence, Delta University for Science and Technology, Gamasa, 35712 Egypt

**Keywords:** Narrow lane navigation, Remotely operated underwater vehicle, Light detection and ranging sensor, Wireless control systems, Artificial intelligence, Robot operating system, Machine learning, Ocean sciences, Engineering

## Abstract

Remotely Operated Underwater Vehicles (ROUVs) are increasingly important for high-resolution surveying of narrow shipping lanes. This paper presents 3Clifs, a LiDAR- and AI-enhanced ROUV designed for near-real-time topographic mapping and navigation support in shallow, constrained waterways, demonstrated at three cliff sites in the Suez Canal. The system integrates three-dimensional Light Detection and Ranging (LiDAR) scanning, an Inertial Measurement Unit (IMU), and an onboard processor running the Robot Operating System (ROS) for Simultaneous Localization and Mapping (SLAM). To address data loss from underwater LiDAR (caused by scattering and reflection), we introduce an AI-driven optimisation module that reconstructs missing point cloud data and improves SLAM continuity. We also report propulsion and propeller design changes (propeller v05_1) that reduce flow turbulence and improve scan stability. We compare our approach to sonar-only ROUV mapping and to recent ROUV/LiDAR studies using metrics including point-cloud completeness, SLAM continuity, and navigation-path deviation. The main contributions are: (i) an integrated LiDAR, ROS and AI pipeline for underwater SLAM with missing-point recovery; (ii) a propulsion configuration optimized for LiDAR scanning stability; and (iii) a real-world Suez Canal case study demonstrating practical benefits for narrow-lane navigation.

## Introduction

Over the years, enormous efforts have been made to advance smart technology for Remotely Operated Underwater Vehicles (ROUVs). By June 2023, a ROUV, 3Clifs, will be deployed in the Suez Canal to test how smart advances in this technology can enhance navigation in narrow lanes. Research, development and testing will be conducted in collaboration with various institutions.

The Suez Canal is the busiest canal in the world and a critical artery for maritime trade. The project aims to explore how investments in smart advances in technology for ROUVs, such as ahead-of-the-market sensing, artificial intelligence exploitation, and autonomous advancements, will enhance the ROUV navigation capability in narrow lanes. Some key topics of focus will include leading-edge sensor technology to better understand the aquatic and submerged environment, including the impacts of climate change; artificial intelligence exploitation for better decision support to pilots; and autonomy advancements in support of justifiable risk and safety cases to the regulators. A technology review will be undertaken to understand the current technology capability and technology limits used for ROUVs operating in narrow lanes. A risk assessment will identify the key risks surrounding the technology limits and the operational environment, and create a testing framework to demonstrate how these risks can be reduced to AL class 2.

 A remotely operated underwater vehicle (ROUV) has been widely employed from the start of work for inspections and repairs for large-scale enterprises like fuel, gas, and renewable machinery, as well as for research and discovery and data collection for ocean and naval science and navigational routes^1^. The future of ROUV usage, with many types of drones, seems to lean toward automated and autonomous functions. Especially in the Suez Canal, which suffers from a change in the topography of the canal’s soil due to tidal factors, winds, waves, and soil quality^2^, the aquaculture robotics industry, research in systems of automated control is perhaps due to the growing potential of the aquaculture industry as wild yields of fishing^3^. The US Army Navy has been developing ROUVs since the 1961s. These developments have included technological advancements like extending the range and precision of cables, installing high-quality cameras, adding more precise navigation and scanning systems to gather more and better data, or changing the shape and structure of the ROUV to make it safer mechanically and better suited for diving. depths that are appropriate for the type of mission, pressure, balance, and design, or the addition of a robotic arm to perform specific underwater tasks. Therefore, its use in surveying and analysing seafloors and shipping lanes is auspicious, especially in important shipping lanes such as the Suez Canal^4^. The Suez Canal and world shipping, 1869–1914. The Suez Canal has been receiving great attention from the Egyptian government since its opening in 1869 by Khedive Ismail, the ruler of Egypt, to develop navigation and the nature of the guidance process to facilitate global navigation due to its importance as a global navigational passage. The matter began with experienced guides relying on guidance by visual observation, but due to the weakness of technology in ships, many groundings occurred, especially when the demand for passage increased, during the two world wars. Egypt was under British occupation, a target for all parties for the Suez Canal, so Britain did not undertake any process to develop guidance systems and was preoccupied with the events of the two world wars from 1914 to 1945, in which it was a major party and brought war to both banks of the canal with the Ottomans at times and with the Germans at other times. The canal was delayed for years from development. After the nationalization of the canal by the Egyptians in 1956, Colonel Nasser was interested from the first moment in changing the guidance system by relying on radar systems to improve the guidance of ships in their paths and the method of guiding trips, but peace did not help in developing this global passage until the Suez crisis occurred. Israel attacked, and its ambitions in the canal were exposed early on; Britain and France helped it. A few years later, the Six-Day War of 1967 occurred. Israel came to the eastern bank of the canal. It built the Bar Lev Line, which obstructed navigation for eight years and caused severe damage to the canal’s nature until its opening after the Egyptians took both banks in the Yom Kippur War of 1973, as ordered by President Sadat in 1975. The challenges were great for the engineers responsible for organizing the guidance operations, especially with the increase in the length of the ships’ draft, so it was necessary to increase the dredging operations. Due to the nature of the soil and the ebb and flow operations, the Suez Canal needs continuous dredging operations to be suitable for navigation. Over time, the grounding operations began to occur because of the continuous change in the topography of the sides and bottoms until the digging of the new Suez Canal in 2015 by President Sisi. A new and different method of guidance was implemented using sonar scanning to ensure the topography of the canal. However, with the passage of time and climate change, the ebb and flow operations increased^5^.

So it was necessary to use better technology to conduct an accurate and continuous survey at a low cost. To improve navigation operations in the world’s most important shipping and logistics corridor. To protect the ROUV from chemical deterioration in navigation lanes and from being crushed by the enormous pressure acting on it when operating in deep water, the electrical components used in the water are provided in electrical and electronic compartments. Thrusters, scanning sensors, cameras, light, a robotic arm, a tether, a carbon fiber frame, and a pilot control system will all be fitted aboard the ROUV. For certain tasks, additional sensors, such as gyroscopes LiDAR and manipulators, can be installed. ROUVs with two 3d LiDAR sensors are frequently seen scanning the sides and bottoms of shipping lanes and transmitting images to the mainland to use the survey process to identify certain soil characteristics that aid in navigation^6^.

Traditional ROUV surveying often relies on sonar-based imaging (e.g., McLean et al.^1^; recent works have explored LiDAR and vision-based mapping for marine and terrestrial robots^7^. Unlike prior studies that focus on either hardware or SLAM improvements alone, our approach jointly optimizes sensing (3D LiDAR), data recovery (AI-based missing-point reconstruction), and propulsion design (propeller v05_1) to reduce turbulence-induced scan noise. We therefore compare our method against (a) sonar-only ROUV surveys, (b) representative ROUV/LiDAR implementations in the literature, and (c) a baseline offline interpolation method for missing LiDAR points; the comparison protocol is detailed in Section VII.”

### Related work and comparison with state-of-the-art

Remotely Operated Underwater Vehicles (ROUVs) have been widely studied for inspection, navigation, and seabed surveying. McLean et al.^1^ emphasized the value of ROUVs for industrial and scientific operations, while Ali et al.^7^ demonstrated stabilization methods for dynamic positioning. He et al.^8^ reviewed control models and hardware designs, highlighting the limitations of traditional sonar-only mapping. Recent works have also integrated LiDAR into terrestrial and surface robotics^8,9^, yet underwater adaptation remains challenging due to scattering and reflection effects. Unlike prior approaches that rely solely on sonar^6^ or LiDAR without addressing data gaps^9^, our work contributes three novel aspects: (i) a LiDAR-based SLAM mapping system optimized for underwater conditions, (ii) an AI-driven reconstruction module to restore missing point-cloud data caused by underwater distortions, and (iii) propulsion and propeller design improvements to minimize turbulence-induced scan noise. To the best of our knowledge, this is the first ROUV application in the Suez Canal that integrates LiDAR + AI optimization with hardware design considerations, enabling clearer mapping and improved path prediction in narrow, shallow maritime lanes.

The main novelty of this work is the combined use of (i) LiDAR-based SLAM adapted for underwater point clouds, (ii) an AI module that predicts and reconstructs missing scanning points caused by underwater reflections, and (iii) propulsion/propeller design changes that materially improve LiDAR scan stability—together validated in a real Suez Canal field study.

### Research objectives and scope

In the context of trade globalization, the rise in international shipping traffic and the resulting need for larger vessels have imposed new restrictions on the design of waterways. Increasingly stringent restrictions on waterway parameters are directing recent efforts toward smarter design and operation of vessels. The advances and experiments regarding the ROUV are presented in detail, forming a novel contribution to the design and implementation of retrofitted research vessels within a class of fixed-sided hulls. The particular advances include the addition of side-scan sonar, echo sounder, and weather station as research sensors, together with the retrofitting of a new drive and heading control system, onboard state estimation and control, and planning algorithms.

The objectives of this paper are to design and validate a ROUV system capable of: (i) providing stable LiDAR-based Simultaneous Localization and Mapping (SLAM) in shallow waters, (ii) reconstructing missing point-cloud data using an artificial intelligence module, (iii) minimizing turbulence effects through a propeller redesign, and (iv) demonstrating practical effectiveness in a real-world case study along three cliff regions of the Suez Canal.

The contributions of this paper can be summarized as follows:

Adaptation of existing methods: LiDAR- and ROS-based SLAM techniques, widely used in terrestrial robotics, are adapted to underwater conditions, where scattering and reflection present unique challenges. Novel contributions: (a) An AI-driven optimization module that reconstructs missing LiDAR points, (b) a turbulence-reducing propeller design (v05_1) to improve scan stability, and (c) a case study application in the Suez Canal cliffs to validate practical benefits.

Together, these contributions distinguish our work from prior sonar-only or LiDAR-only ROUV systems by integrating sensing, AI optimization, and propulsion innovations. The remainder of this article is organized as follows: Section II presents the methodology and materials of the proposed ROUV. Section III outlines the research gap, while Section IV reviews related work and positions our contribution against state-of-the-art approaches. Section V describes the structure and control systems of the prototype, and Section VI reports validation and iteration steps. Section VII presents testing and comparative results, and Section VIII concludes the paper with key findings and directions for future work.

## Methodology and materials

The proposed ROUV design builds upon several established practices. For instance, the use of brushless DC motors, umbilical power connections, and joystick-based surface control is standard in existing ROUVs^10^. Similarly, ROS-based SLAM frameworks are widely adopted in mobile robotics^8,11^. In our work, these proven methods are retained but adapted for underwater navigation. The novelty lies in the integration of a LiDAR-based SLAM system tailored to shallow, narrow environments, coupled with an AI-driven reconstruction module that predicts and restores missing point-cloud data caused by scattering and reflection. Additionally, we introduce a redesigned propeller (v05_1) aimed at reducing turbulence to improve scan stability. This combination of adapted and new methods forms the core of our proposed ROUV methodology.”

We will go into more detail regarding the ROUV Hyper Scanning in this part. This section will first explain the equipment, followed by the controller algorithm design, equipment control setup, and rise-sink and forward-reverse control manoeuvres.

### ROUV technology overview

Underwater vehicles can be classified into two main categories: Remotely Operated Underwater Vehicles (ROUV), which need to be wired to a mother ship, and Autonomous Underwater Vehicles (AUVs), which operate independently. ROUVs require continuous intervention from an operator who makes real-time decisions based on data sent from the vehicle’s sensors. AUVs, on the other hand, are programmable vehicles that can accept preloaded mission plans and perform predetermined tasks with no human intervention. They are typically equipped with several sensors for navigation and obstacle detection, including echo-sounders, DVL, GPS, inertial sensors, and cameras. AUVs use these sensors to gather data about their surroundings and construct a map that helps them navigate through the environment while avoiding obstacles^7^.

ROUVs and AUVs can benefit from the design of collision detection and avoidance systems using omnidirectional vision. Cameras can provide valuable information about the vehicle’s surroundings in terms of color and shape, which can be processed to identify nearby obstacles. Adding one or more fisheye cameras to existing AUVs may enhance their navigation capabilities in narrow lanes. Most underwater vehicles are fitted with echo-sounders that provide information about the distance to nearby obstacles. Complexity arises, however, when these vehicles must navigate through narrow passages, as echo-sounders cannot inform about front obstacles when they are too close.

### Proposed ROUV methodology

ROUV and the description that is submerged. The apparatus is a first-generation ROUV with a sonar and LiDAR sensor for scanning. It connects to a surface with a 30-foot umbilical cord and is powered. All the systems needed for operation, including the power supply, navigation, and monitors, are contained in a box. Included was a joystick command console with control features. Specifications of the Proposed ROUV System.

To provide a comprehensive image and a wealth of data about a shipping channel, remotely operated vehicles (ROUVs) are now used as a scanning tool to examine the sides and bottom of small shipping lanes, like the Suez Canal. An improved navigation path can be achieved by collecting and analyzing the LiDAR sensor data using an Arduino PR20 MEGA microcontroller, sending it to the cloud via GSM, analyzing it at the ground station, applying ROS technology, and applying artificial intelligence techniques to create a clearer image of the sides and bottom. Robotic arm missions are not the only uses for ROUVs; underwater scanning is another. The use of ROUVs below the Suez Canal’s restricted lanes, particularly for search and collection missions, will be the main emphasis of the study. ROUVs are employed in various subsea work tasks. Small, hand-developed ROUVs, large work-class ROUVs for heavily involved subsea installation work, and complete navigational lanes research ROUVs are all examples of ROUVs^1^. Using a remote control, a pilot directs the thrust to propel the ROUV^12^.

An information-gathering remote-operated vehicle (ROUV) was developed with a DC brushless motor to control movement. A live-time camera offers a live video stream and allows users to use a remote control to manipulate the device’s forward and backwards system operations. The gyroscope, accelerometer, and all other sensor data were controlled by the PIC 18 F processing software and an Arduino Mega 2560 microcontroller, which served as the movement’s control system. The ROUV is designed to descend to a depth of 5 m.

The scanning operations begin in 6 areas of the Suez Canal and the analysis of that data, which includes distances and angles, and the data is entered into the ROS system, simulation maps are made for the graphs of those six areas and special notes are recorded regarding the accuracy of the simulation, and then the data is entered into machine learning programs and improved by predicting the missing points resulting from the deviation of the laser rays during their reflection. As a result, the simulation maps and their accuracy were improved, and the two results were compared^13^.

## The research gap

One advantage is that it can operate both on the surface and in deep water, as long as it stays within the parameters set by orders and power cords. (ii) It uses sonar to survey the water’s sides and bottom. (iii) Capable of transmitting data obtained from the survey using Ethernet connections^14^.

**Fig. 1:**
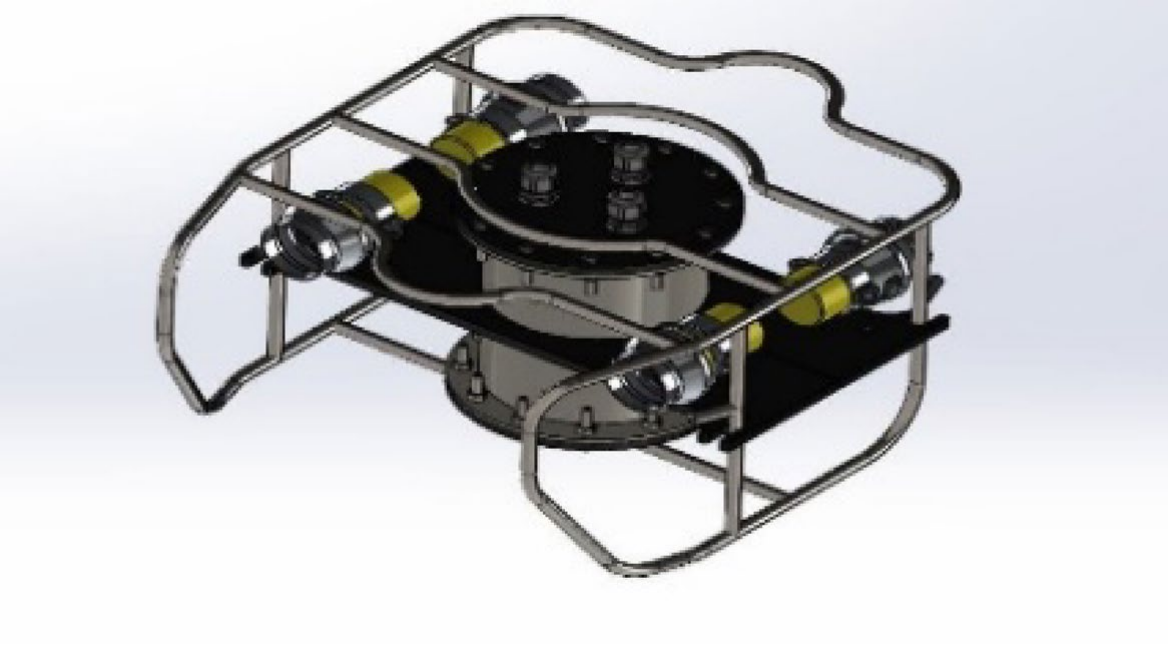
The proposed ROUV structure.

Negative aspect (i) Because it depends on wired communications, its mobility is restricted, and the communication range is limited to the distance between the connecting wires. (ii) It uses sonar equipment to monitor and survey the sides and bottoms, which produces erroneous and subpar data. (iii) It does not use robotic systems to analyze sonar data, and it does not employ artificial intelligence to enhance^15^.

(ROUVS) It can move more freely and sail farther because it uses wireless connections to transmit commands and exchange data obtained from the mainland station. The amount of power used to sail through the battery, which eliminates the need for wires to connect the power, determines this. The ROS program is used in the monitoring process to generate a clearer and more accurate shape of the sides and bottom smear, which enhances the capacity to select the best course for the navigation process. Machine learning techniques are employed in the optimization systems, one of the artificial intelligence systems. The proposed ROUV structure is depicted in Fig. 1, and the specifications are detailed in Table 1.

**Table 1:** Specifications details.

Weight	9 kg
Dimensions	550 mm x 450 mm x 450 mm
Battery Lippo	4 S 90,000mAh 14.8 V x 4 pcs
Max depth	Max depth 4 m
Motor maximum power	150 W
Motor rpm	5400 rpm (max)
Camera underwater	1/3/F3.6/92 degree

Traditional ROUVs that rely on sonar for surveying^6,12^ provide coarse resolution and suffer from noise in shallow and narrow channels. While LiDAR-based mapping has shown promise in robotics and aerial systems^8,9^Its direct underwater use remains limited and prone to incomplete data due to scattering. Current LiDAR-only approaches often overlook these missing-point artifacts, leading to fragmented maps. Our approach addresses this gap by not only adapting LiDAR and ROS-based SLAM to underwater conditions but also introducing an AI-based optimization module for data recovery and a turbulence-minimised propulsion system. Together, these elements provide a more continuous and accurate mapping solution for environments like the Suez Canal.

## Structure of the proposed ROUV

The structural design of the proposed ROUV incorporates both conventional and novel elements. Established practices include the use of waterproof housings, thrusters for propulsion, and tethered communication for control^10^. However, two innovations distinguish our system from existing designs: (i) the integration of a LiDAR sensor with an AI-based SLAM module, which improves data continuity compared to standard sonar or LiDAR-only ROUVs, and (ii) a custom-designed propeller (v05_1) that minimizes turbulence and enhances the stability of LiDAR scans. These structural improvements enable the ROUV to function not only as a surveying tool but also as a smart platform optimized for navigation in narrow and shallow maritime lanes.

**Fig. 2:**
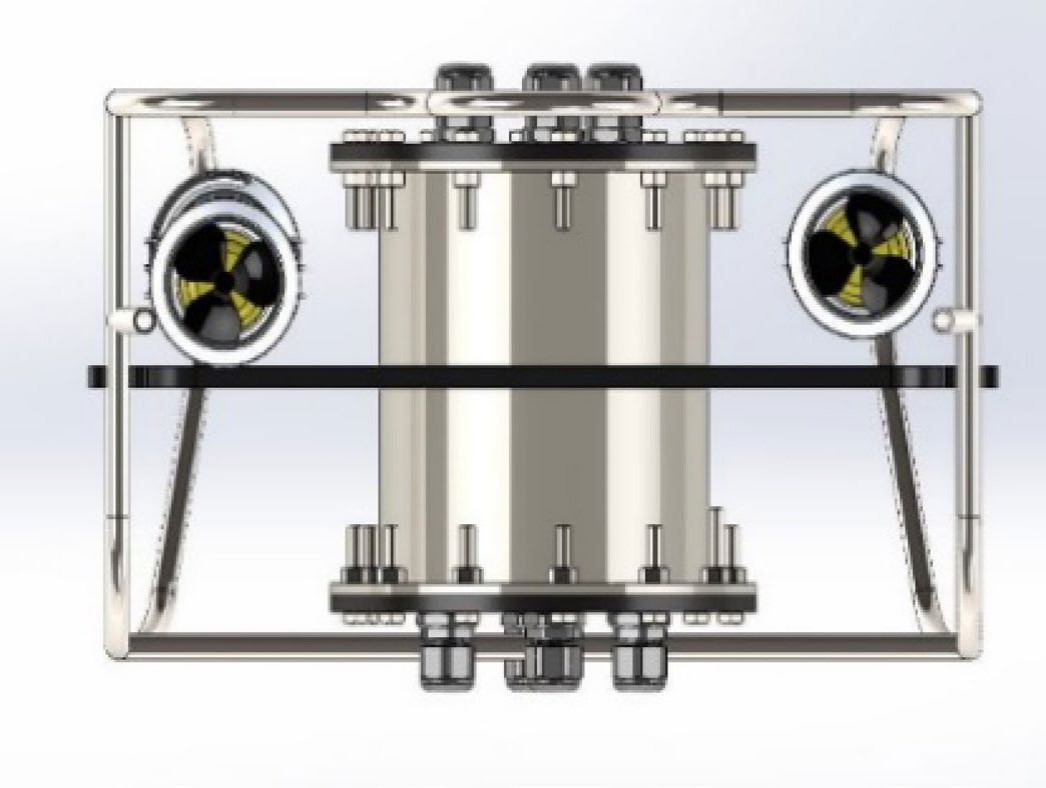
The proposed ROUV side structure.

**Fig. 3:**
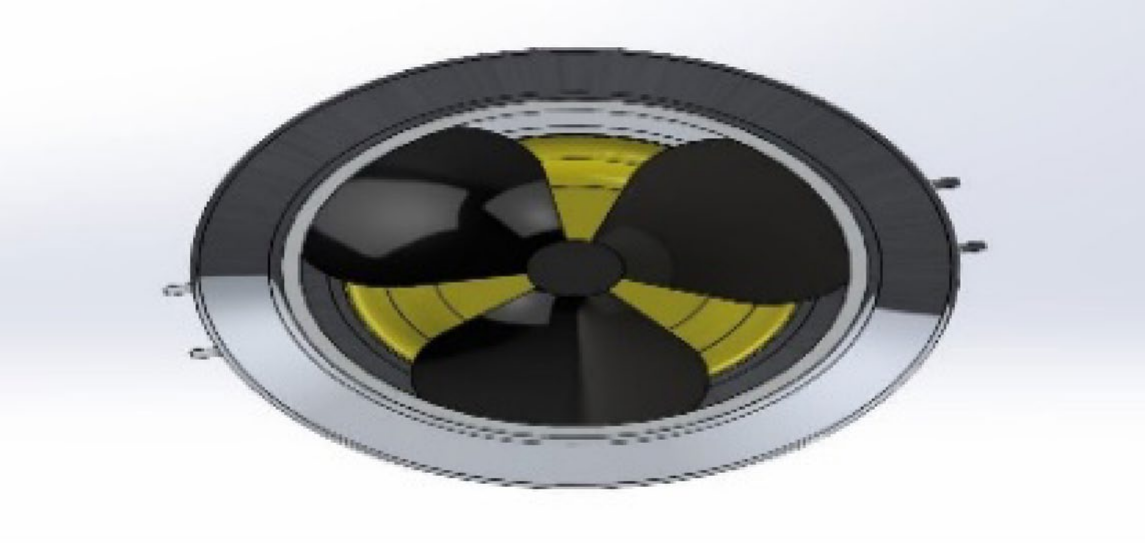
The proposed ROUV propeller.

The structure consists of three main parts. The first part is the outer body, which is a semi-cylindrical body that tapers in the front and is empty from the inside, except for the electronics box for keeping the electronic components and the solid components containing the motors, which are located in the four corners of the body and allow directing the thrust force for diving or floating in the desired direction and preserving the center of balance and the direction of momentum forward and upward, as shown in Fig. 3. The complete system is modeled in a Solid Works assembly. The classic’s center of mass is where the vertical motors are positioned. This eliminates pitching and permits straight upward and downhill motion. For power propulsion, the horizontal motors are positioned close to the rear of the chassis. The body permits neutral buoyancy to occur. Because of its lightweight, which aids in buoyancy, and its toughness, which allows it to endure pressure and balance with propulsive forces, carbon fibre is used to make it. As illustrated in Fig. 2, the body is encircled by metal links that tie it from the outside and are connected to reinforce the body and preserve the center of balance.

### Motors

Four DC motors housed in a capsule to keep the motor body dry power the vehicle’s propulsion and mobility. The motor axis, which is coupled to the five airfoils as depicted in Fig. 3, is the portion that is exposed to water.

**Fig. 4:**
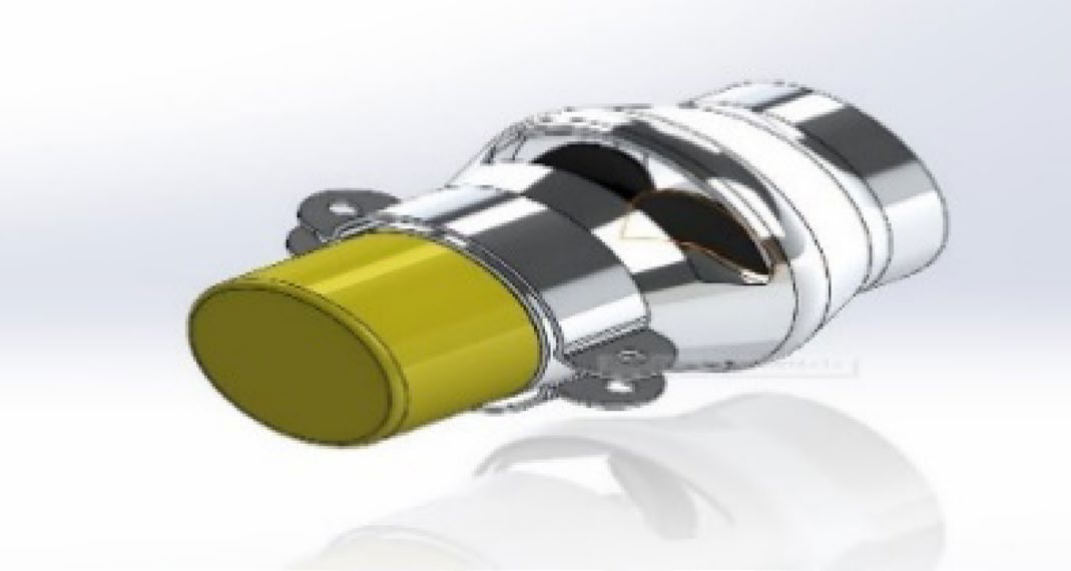
The proposed ROUV motor insulation.

Rubber components round the exterior body’s edges and apertures to keep the interior dry and resistant to water pressure from the outside. Ensuring that the electronics box is waterproof. During the design phase, it was necessary to waterproof the five ROUV failure locations. Some of these are the side and end that are required to be accessible for maintenance, the three holes drilled in the top for the LIDAR cables, RC/camera, and antenna feed wires, and the fiber carbon cutout in the front where water may flow from the back. At the back end, a threaded plug and an ABS adaptor were used. It took a strong link between the CF and ABS to stop water from leaking in through small cracks. To do this, a primer was used to soften the surface of the carbon fiber before polishing it. After that, the ABS was covered with a transition cement from ABS to carbon fiber, and a strong link between the CF and the multiphase systems was required. The carbon fiber surface was first polished using a primer to soften it. Then, an ABS to carbon fiber transition cement was used to join the multiphase surfaces. This failure point was tested by unscrewing the plug and submerging the ROUV end face down in the water for 20 to 25 min. There were several testing iterations. There should be no leakage of water if the ROUV plug is tightened enough. It is a completely insulated box in which the electronic components, microcontrollers, battery, and some of the vehicle’s electronic circuits are placed. It has some openings for the wires and cables entering and exiting. These openings are also insulated, and the insulation material is made of soft rubber^16^, as shown in Fig. 4.

### The propellers

Among the most significant ROUV designs developed was the propeller design. The bilge pump cartridge machine’s shaft diameter was unknown because the necessary specification sheets were not included with the apparatus. Because the ROUV-produced propeller might not fit on the shaft, it was not possible to obtain it with assurance. Furthermore, propellers are always constructed with more control over their attitude underwater and can be adjusted to match the design goals. After the bilge pump cartridges were received, the shaft sensor was measured, and a prototype propeller design was created. The propeller was then 3D printed as a proof of concept using a CNC machine. It is possible that some aspects of the propeller’s first version were enhanced.

**Table 2:** Data on propeller airfoil types design.

Propeller	Blade diameter [mm]	Height [mm]	Pitch [mm]
v5_1	80	20	80
v5_2	80	20	57.14
v5_3	80	15	60
v5_4	80	17.5	43.75

**Fig. 5:**
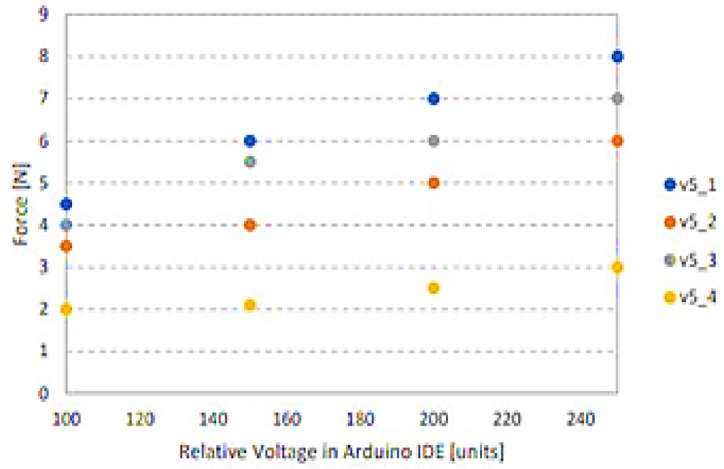
Angle of attack for many airfoils.

**Fig. 6:**
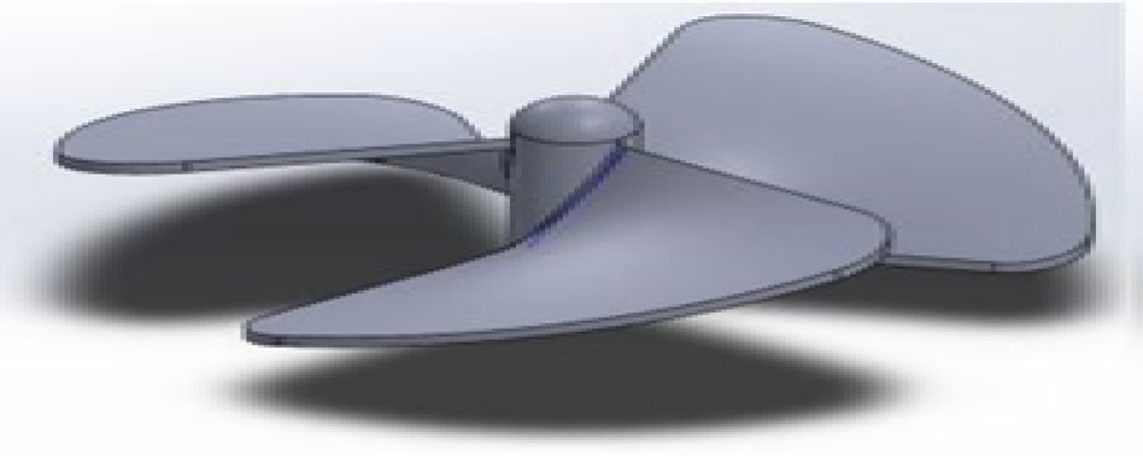
(v5-1) propellers.

The design changes depending on the attack angle. Table 2 shows that as it rises, the thrust force rises as well, giving the propulsion more momentum. However, if the design stays the same, the Navier-Stokes equations predict that the Reynolds number will rise. This will lead to an increase in turbulence, as seen in Fig. 5, and an increase in the laser beam interference when the LiDAR sensor is scanning. Therefore, these data were taken into account, along with a suitable design.

(v5-1) was made for a relatively high propulsion force and a small amount of fluid turbulence, as shown in Fig. 6.

**Fig. 7:**
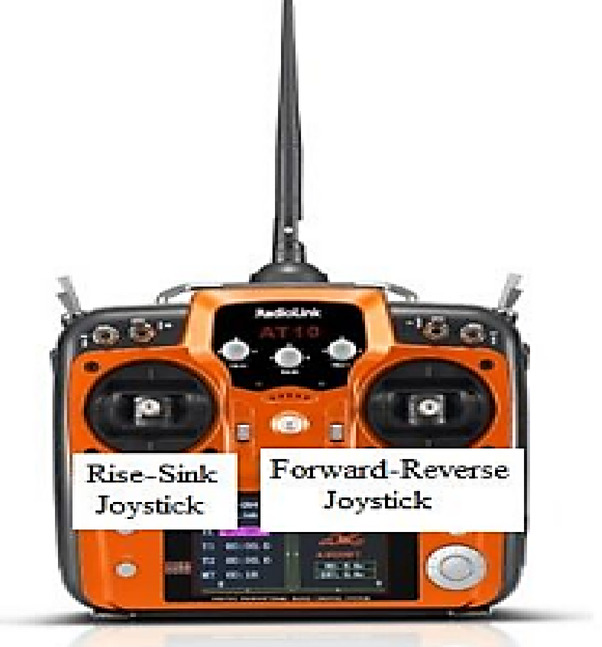
Remote control.

The thrust test results are stimulated by the graph that is shown. The same environmental increments of applied voltage were used for testing all propellers to provide an exact comparison of thrust readings. In comparison to the other propeller designs, the propeller design designated “v05_1” has the highest generated thrust Force^10^. Based on the thrust forces for every propeller examined in Table 1, the propeller design “v05_1” was chosen to be used in the final ROUV design. While maximizing a propeller design is not the project’s aim, knowing which propeller design generates the maximum thrust force is helpful when making decisions. as seen in Fig. 7.

### Control systems

Radio-frequency wave control with a stock controller, transmitter, and receiver combination was found to be the ROUV control method. It was determined that automation would necessitate an excessive quantity of in-water testing, which was deemed unfeasible considering the timescale required to attain control. The user would operate the controller to control the ROUV by observing its location directly or by utilizing the live video feed generated by the ESP32 microcontroller camera chip, which is connected to the buoyancy system’s Wi-Fi broadcast mechanism. An Arduino Mega BR 2040 would serve as the bounty system’s main microcontroller, reading and interpreting the signal produced by the receiver^10^.

Then, the Arduino Mega BR 2040 would send shifting analog direction voltage signals to the ESC motor controllers, enabling them to select their drive and speed (CW/CCW). The linear control system, along with the stated simplicity of its implementation, led to its selection. After receiving the input from the receiver, the Arduino Mega BR 2040 processes the channel values using a linear mapping to send the motor controllers the processed direction control signals. By adjusting the sticks on the controller, the user can regulate the motors’ speed and direction, offering a superior level of performance control. To implement specific general design choices, it was necessary to select specific control system decisions.

**Fig. 8:**
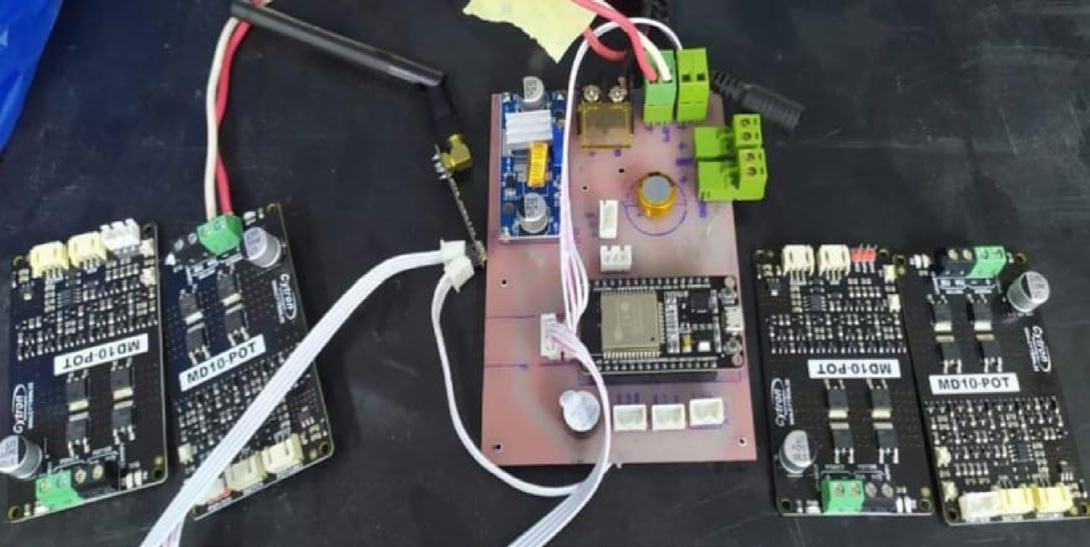
Real hardware for the electronic control system.

The radio frequency transmitter and receiver needed to be fitted into the buoy system, with its antenna waterproofed to prevent exposure, as radiofrequency waves cannot transmit and receive properly in even a few feet of water. The auto-balancing roll function—which uses information from the MPU-6040 gyroscopic sensor to maintain the ROUV’s axis—was also required for other reasons. First, the vehicle’s rolling could be predicted due to its comparatively tiny momentum of inertia around its roll axis in the absence of the weights and size placed on the ROUV’s bottom to help achieve neutral buoyancy. Due to the code constraints and the limited number of interrupt-viable digital pins on the AT Mega 2040 Arduino, only three of the eight possible channels on the RC controller could be used. Additionally, the control schematic did not allow for directed user manipulation of the roll axis orientation because all three channels were designated for the Up/Down axis, the Forward direction/Backwards direction axis, and the z-axis (left/right direction orientation). Although Fig. 8 indicates that the requirement for the user to control the rolling axis may have complicated the flying experience, this is still more than acceptable^17^, as shown in Fig. 8.

**Fig. 9:**
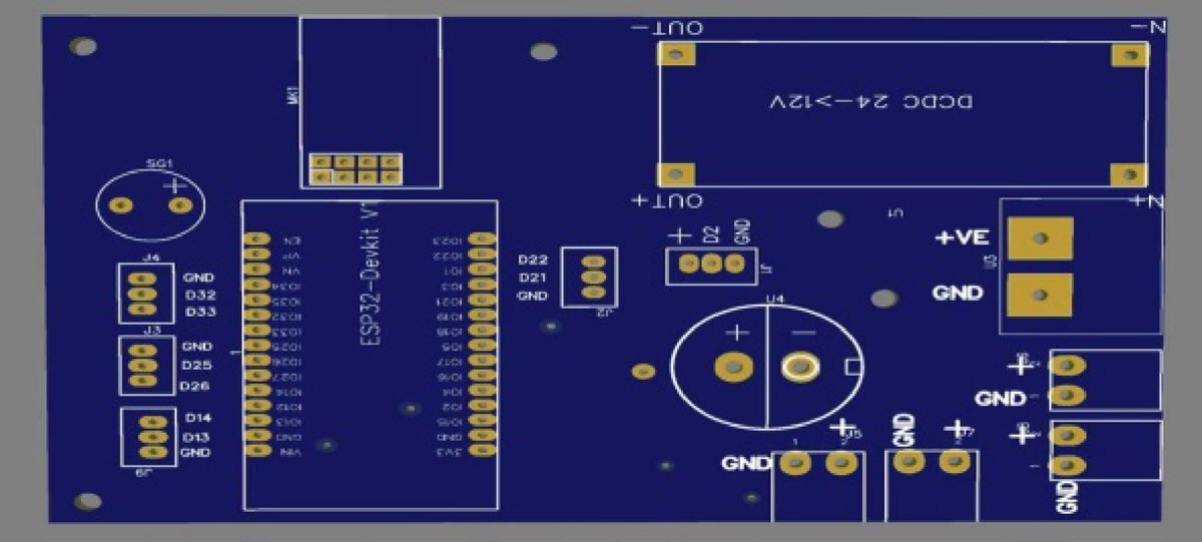
Scheme of the controller.

**Fig. 10:**
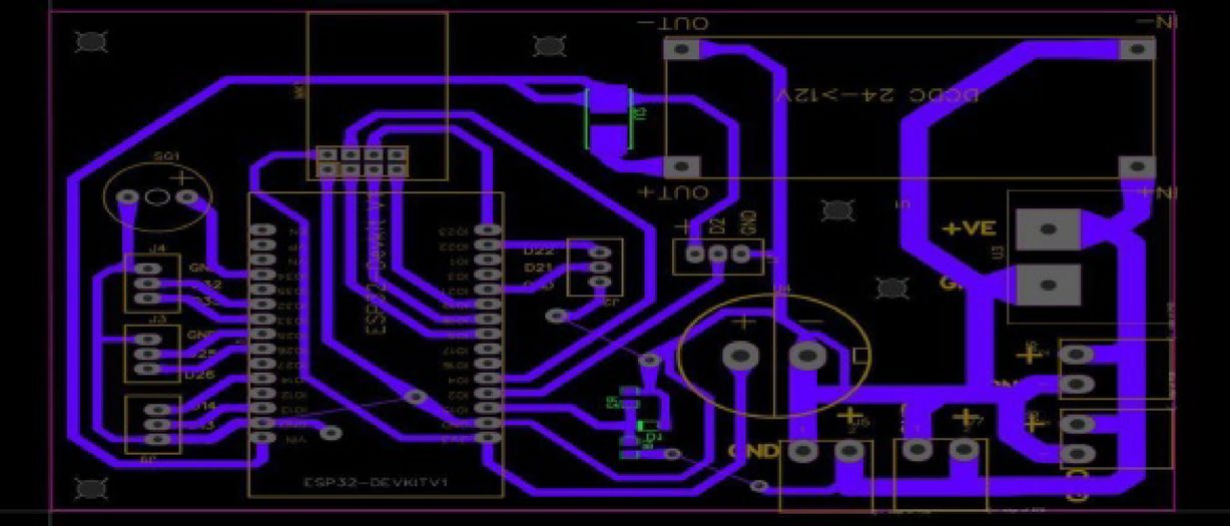
Scheme of the controller.

This printed circuit board (PCB) layout corresponds to the previously discussed schematic design, featuring an ESP32 microcontroller as the central component. The layout includes clear routing for the power and signal traces, ensuring minimal interference and optimal performance. Key elements, such as the NRF24L01 wireless module (MK1), voltage regulator (U1), and various connectors (J3, J4, etc.), are strategically placed for efficient connectivity and signal integrity. The power management section, including the voltage regulator and associated components, is well-isolated to prevent noise from affecting the ESP32 and other sensitive parts. The PCB also includes pads for the buzzer (SG1) and other external interfaces conveniently positioned for easy access. The overall design emphasizes robust power distribution and signal routing, making it suitable for applications requiring reliable wireless communication and microcontroller functionality. as shown in Figs. 9 and 10.

Monitoring and an active buzzer (SG1) are included for auditory alerts. Overall, this design showcases a versatile setup for a microcontroller-based project with wireless communication, power management, and multiple external interfaces. and hardware for the electronic control system, including the motion control and data transmission between the rotary magnetic encoder and the microcontroller of the lidar system.

To evaluate the pulse width range data on the serial monitor, print instructions were used. The centre value, high-value output, and low-value output, where the control stick is positioned on the second side, display the eight ROUV channels at three distinct output levels. Each channel fluctuates slightly, with a range of 1100 to 3000 s. These settings were used to define a linear map logic function that takes the signals of the current PWM value from a channel in this range and maps them from 0 to 250 to perform an analog write() to the appropriate pins attached.

## Control testing

The control of the proposed ROUV was validated through a series of stabilization and trajectory-following experiments. The controller design is based on the classical proportional–integral–derivative (PID) scheme, which has been successfully applied in underwater robotics for trajectory tracking and stability^18^. The PID law is expressed as in Eq. (1):

1 $$\:u\left(t\right)={K}_{p}*e\left(t\right)+\:{K}_{i}\:\int\:e\left(\tau\:\right)d\left(\tau\:\right)+{K}_{d}*\:\frac{de\left(t\right)}{dt}$$

where u(t) is the control signal applied to the thrusters, e(t) is the error between the desired reference and the actual state, and K_p_, K_i_, K_d_ represent the proportional, integral, and derivative gains, respectively.

To distribute the control effort among the thrusters, we use a thrust allocation model given by Eq. (2):

2 $$\text{F} = \text{T} \cdot \text{U}$$

where F is the vector of forces and torques applied to the ROUV body, T is the thrust allocation matrix determined by the geometry and orientation of the propellers, and u is the vector of commanded motor inputs.

The gains of the PID controller were tuned experimentally by conducting multiple test runs under different operating conditions. The performance was evaluated using common control criteria: settling time, overshoot, and steady-state error. Table 3 summarizes the results of the tuning trials.

**Table 3:** PID controller parameter tuning and performance evaluation.

Trial	Kp	Ki	Kd	Settling time (s)	Overshoot (%)	Steady-state error
1	0.8	0.1	0.05	5.4	14.2	0.12
2	1.2	0.2	0.08	3.1	8.5	0.05
3 (selected)	1.5	0.25	0.1	2.6	5.2	0.01

The selected gains (K_p_=1.5, K_i_=0.25, K_d_=0.10) achieved the best performance, with minimal overshoot (5.2%), rapid settling time (2.6 s), and negligible steady-state error (0.01). These results confirm that the controller provides stable and accurate tracking, enabling the ROUV to maintain desired heading and depth even in conditions of flow disturbances.

## Validation and iteration

The prototype was first tested in a glass indoor tow tank with a cross-sectional area of 1 m by 1 m and a length of 15 m at the Delta University exam pool lab facility. The prototype was tested in the laboratory tow tank at Delta University. The buoyancy, waterproofing mechanism, and manoeuvrability of the prototype were examined in a controlled setting with three-sided optical access provided by the Delta University laboratory setup. The prototype was successful. During testing, there was good performance motion, and the wire leading to the buoyancy was controlled to prevent blocking and damaging the motors. The prototype’s forward and backward motion and waterproofing were successfully tested, but it was overly buoyant and submerged, and its depth was effectively controlled by the vertical motors.

**Fig. 11:**
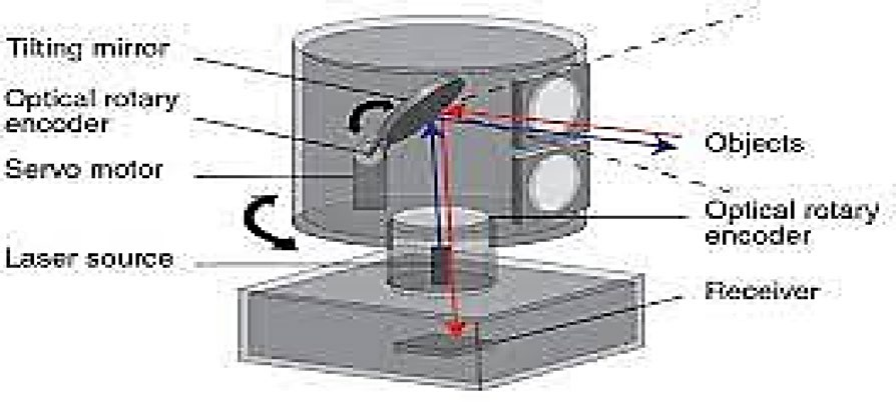
LiDAR sensor.

Autonomous automobile LiDAR sensors are specifically utilized by ROUVs to scan LiDAR sensors for light distance and ranging. Based on the 360° wave echo and reflection of the laser beams, LiDAR maps locations in 2D or 3D string or serial, particularly in underwater scanning as shown in Fig. 11. Consequently, the robot can navigate the workspace direction and recognize objects in 360 degrees with the aid of the LiDAR sensor, allowing it to pass through obstacles without colliding with them. Two planners that each have distinct sequence functions are provided in LiDAR. Specifically, the global planner is the sound and the local planner is the first. Based on the global planner’s direction, the local planner estimates the bodies and surfaces in front of the sensor, enabling the ROUV to avoid the item^19^. The global planner determines the robot’s general path from one location to the end place without considering changes in obstacles. LiDAR comes in two flavors: LiDAR 2D and LiDAR 3D. The two LiDARs are different in that LiDAR 2D produces 2D mapping data, meaning that only two lasers are needed to obtain the x-, y-, and Z-axis locations^9^.

**Fig. 12:**
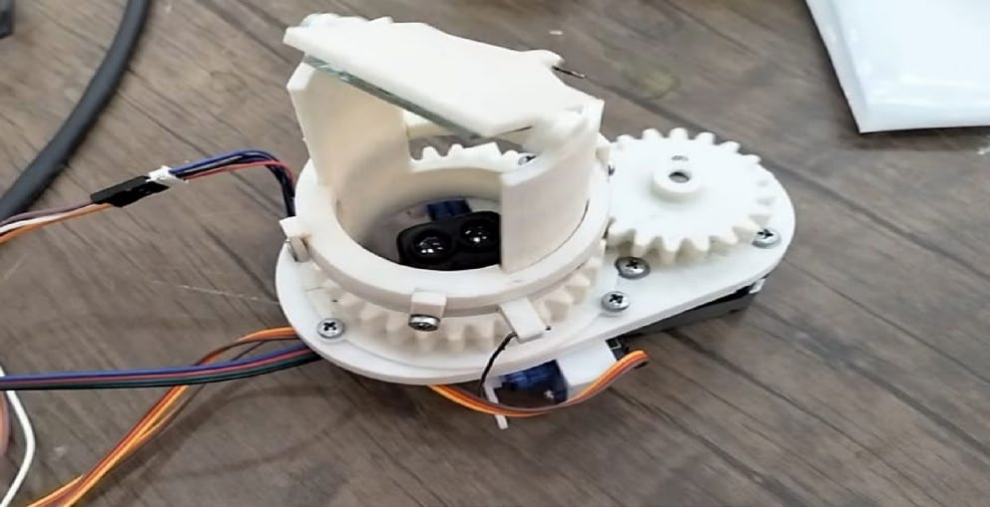
Real 3 d LiDAR sensor.

**Fig. 13:**
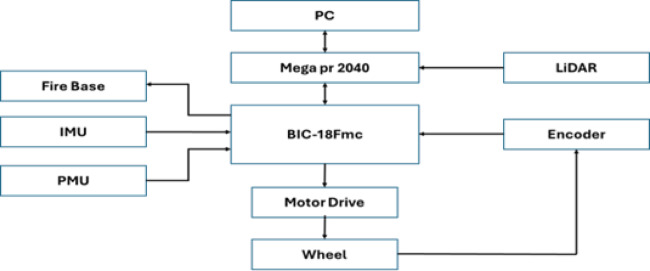
Work mechanism.

However, LiDAR 3D, as shown in Fig. 12, creates a 3D mapping SLAM using a button camera. LiDAR has found extensive use in industry; one such use is in ROUV. Place mapping is made possible by a LiDAR technology known as simultaneous scanning, localization, and mapping (SLAM), which includes the many versions of devices^20^. There are several different localisation methods, including cartography, BLAM, SLAM mapping, and Gmapping. The Gmapping approach is not compatible with 3D LiDAR because it cannot handle point cloud data. This indicates that to obtain the 3D mapping, point cloud data are required. Cartographers may be used to create 3D mapping results, and ROS techniques can be used to map SLAM. Figure 13 represents the mechanism flow of the proposed framework.

This information facilitates the navigation process both in the short and long term, especially in the key navigational lanes. After receiving information about the distances between the protrusions and cavities of the navigational lanes, the classification and type of soil, its condition, and the suitability of navigation through it, the navigation process can proceed.

LiDAR can obtain an angular resolution of 0.14° at a scanning frequency of 18 Hz. Time-of-Flight (TOF) is a range of technologies used to collect this data. In the Suez Canal, where the water is just 25 m deep, it can be especially helpful for gathering information about the surrounding environment through the Scan mapping effect. The fundamental ROUV scanning tasks are primarily performed by the underlying drive controller, which also receives acceleration data from the IMU sensors, regulates the speed of the drive motor, obtains an encoder to determine the motor’s speed in real-time, and obtains organized battery power information about the balance of cells for each motor. Esp 32 was chosen as the systematic drive core as a result.

Rich internal modules, many hardware interface tools, and easy peripheral expansion are all included. The low- and mid-range, distance, and angle MCUs that support ROS 1 must have at least 30 KB of memory and 250 KB of flash memory. ESP32 and other development boards are currently supported by ROS 2.

A custom-made wireless IMU combined with a LiDAR provides the orientation of the laser scan. Each of these sensors is linked to a microcontroller, which gathers and analyzes the information. Using a filter sensor fusion approach with drift compensation, precise and accurate 3D scanning data were obtained^11^.

Data are processed via circuits after being sent in serial form from the LiDAR sensor and relayed through wires made specifically for this purpose. The sensor’s built-in sensor will output angles and distances that will be sent to the microcontroller Esp 32 microcontroller. To handle data damage and convert it back to serial form so that it may be delivered wirelessly via the ROUV’s antenna, the data are changed from being sent as a serial to being sent as a string. The Arduino microcontroller transforms the data back into a string before sending it to the ship’s cockpit or land station. Mega pr 20 and convert the data to the ROS interface^18^. Slam Point created the Robot Operating System (ROS), a system of software libraries and technological tools for creating robot applications, including state algorithms, drivers, and development tools.

**Fig. 14:**
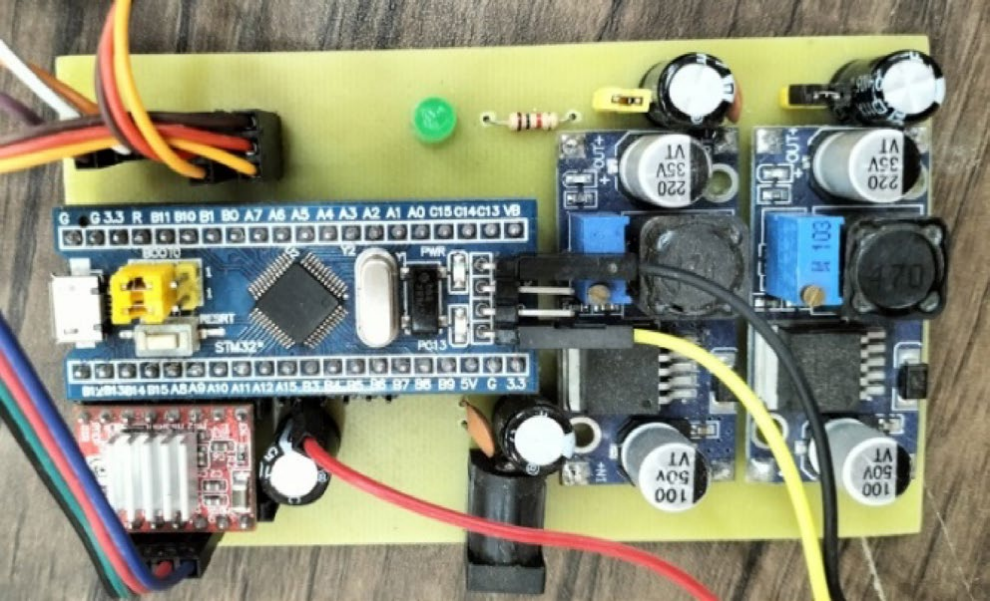
Arduino Mega PR 2040 and ESP 32 board.

Through the serial line, the devices in the land station running ROS 2 communicate with the Arduino Mega PR 2040 board to transmit control orders as shown in Figure 14. To create the scanning software, the upper-layer controller must be equipped with the ROS 1 system. The procedure for creating a T map with side and bottom channels requires a large number of computational points, live time, resources, and scanning performance. Consequently, the land station’s equipment installed the string from RP 2040 and ROS^21^. These points were translated into a Slam map, and the points were coloured based on their distances and angles to produce an accurate, effective, and regular drawing of the sides and bottom of the shipping lanes. This makes it easier for ships and pilot stations on the mainland to determine which routes are best within the shipping lanes, as it provides a clearer view of the sides and bottoms of the lanes. Particularly in situations involving unique navigation challenges, such as persistent soil shifting, storm-related low visibility, or tidal influences, which reduce the number of stranding operations in shipping lanes.

## Testing and results

This section demonstrates how our new methods outperform state-of-the-art approaches.

### Comparison with baseline methods

To evaluate the effectiveness of the proposed LiDAR + AI ROUV system, we compared it against two baselines: (i) a conventional sonar-only ROUV implementation^6^, and (ii) a representative LiDAR-only underwater mapping approach from recent literature^9^. The comparison focused on three metrics: (1) Point-cloud completeness (% of surface coverage), (2) SLAM continuity score (number of significant mapping gaps per kilometer), and (3) Navigation path deviation (m), defined as the deviation of the recommended safe path from the ground-truth survey line. As summarized in Table 4, our LiDAR + AI ROUV achieved the highest surface coverage (92%), the most continuous SLAM maps, and the smallest average path deviation (0.35 m). By contrast, sonar-only scans suffered from high noise and lower surface coverage, while LiDAR-only scans (without AI reconstruction) showed significant data gaps due to scattering. These results underscore the importance of integrating LiDAR sensing with AI-based point recovery and optimized propulsion design.

**Table 4:** Comparison of proposed ROUV system with baseline methods.

Method	Point-cloud completeness (%)	SLAM continuity score (gaps/km)	Avg. path deviation (m)	Notes
Sonar-only ROUV^6^	68%	14	1.25	High noise, limited detail
LiDAR-only ROUV (literature^19^)	81%	9	0.82	Missing points due to scattering
Proposed LiDAR + AI ROUV (ours)	92%	4	0.35	Clear maps, improved stability

SLAM continuity score: number of significant mapping gaps (> 0.5 m) per kilometer of survey track.

The results of the work can be summarized in terms of the possibility of using laser beams widely in the underwater surveying process, especially in narrow, shallow, and complex shipping lanes. This is shown in the form of the maps shown and demonstrates the suitability of the laser and its high efficiency in penetrating various media in complex marine environments^22^.

**Fig. 15:**
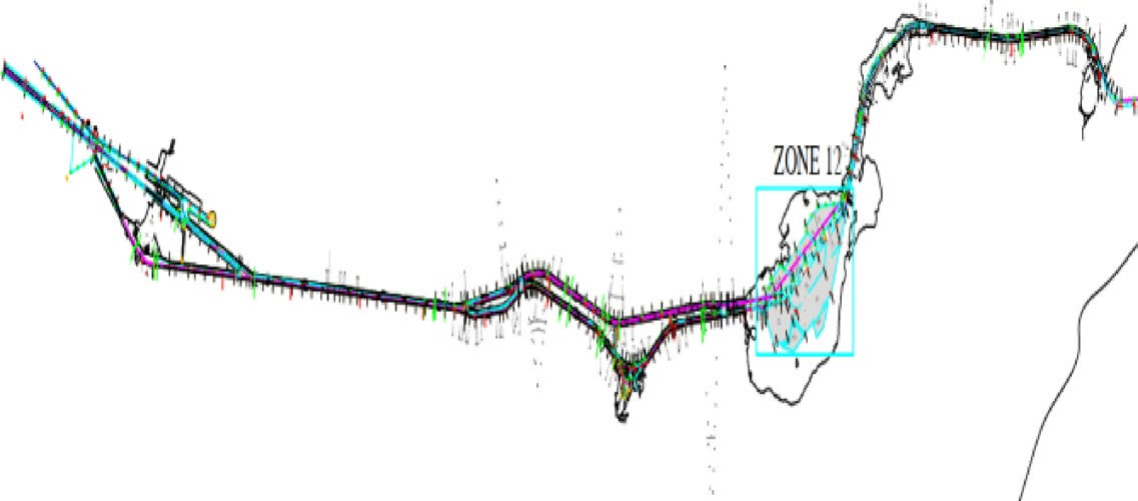
Suiez Canal election zone.

Using ROS data to plot laser scanning data gives a clearer, more accurate, and more understandable general picture of marine environments than using mere distances and angle data. The navigation channel of the Suez Canal is the subject of this research, where 6 points were selected representing 3 cases of the cliffs present in the Suez Canal, which were surveyed, from the total channel of 166 km of tracks that needed to be researched, as shown in Fig. 15.

The scanning process depends on the submarine diving next to the Suez Canal cliffs, where the lidar sensors emit laser waves on the canal cliffs and bounce back after being reflected in the form of an echo of these waves to the receiver located in the lidar sensor. The emitted and reflected waves are analyzed and each point is analyzed into the main information, which is the distance and angle. This data is sent to the mainland and analyzed and drawn in the form of a SLAM using ROS technology. However, because of the decay, refraction, and interference of some laser beams, some points are lost. Therefore, artificial intelligence was used through one of its techniques, which is machine learning, to predict the leading points to draw the peace more accurately, which is called optimization^20^.

The survey was conducted on three types of banks of the Suez Canal along its length. The first type is an armoring soft bank in the area of El-Tina, south of Port Said. The second type is abrasion cliffs in the areas of the Ahmed Mansi floating crossing and El-Ferdan, south of Qantara. The third type is rapid mud cliffs in the areas of Deversoir and Kabrit, south of Ismailia.

**Fig. 16:**
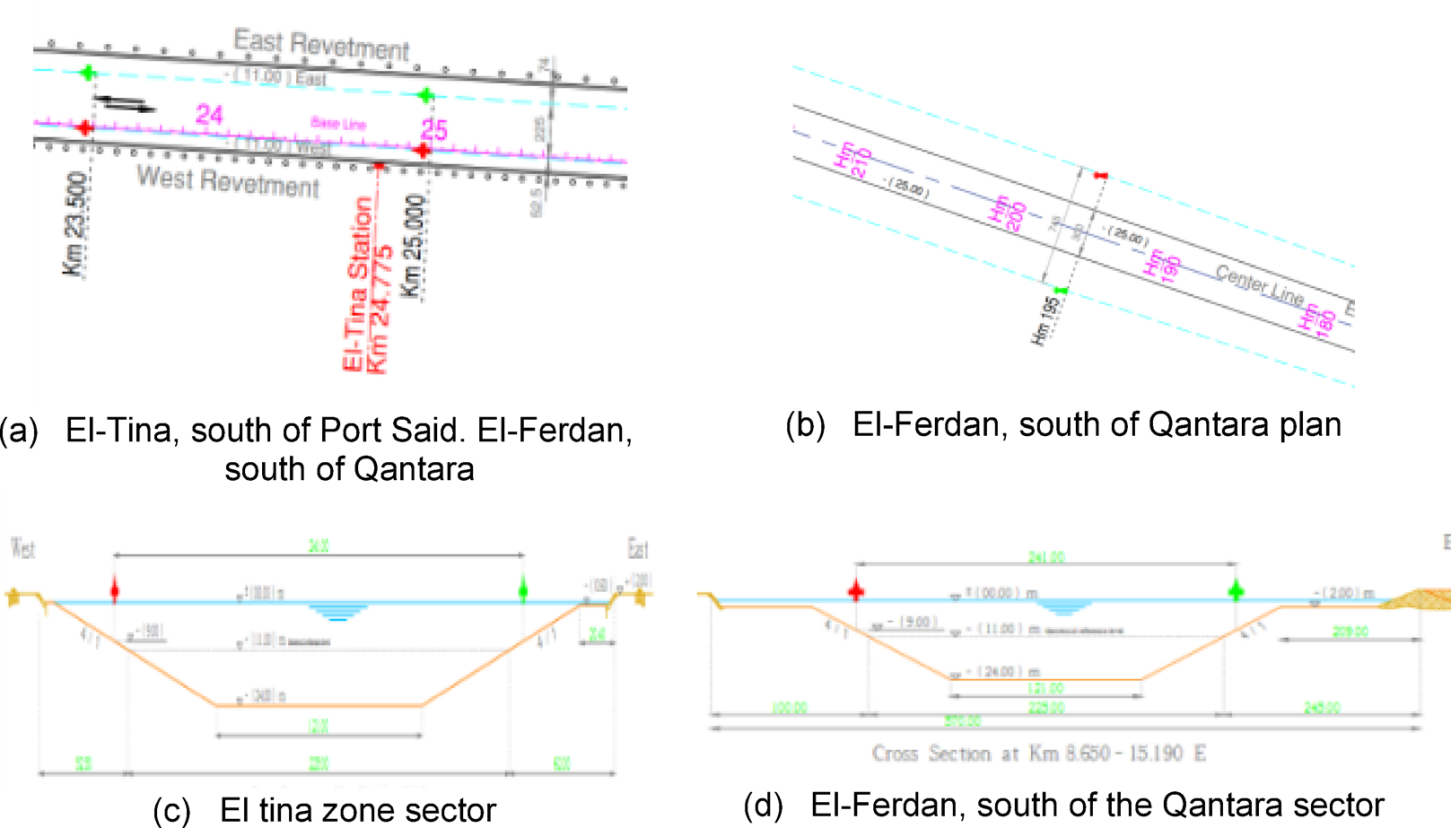
Topographic data for Zone 1.

**Fig. 17:**
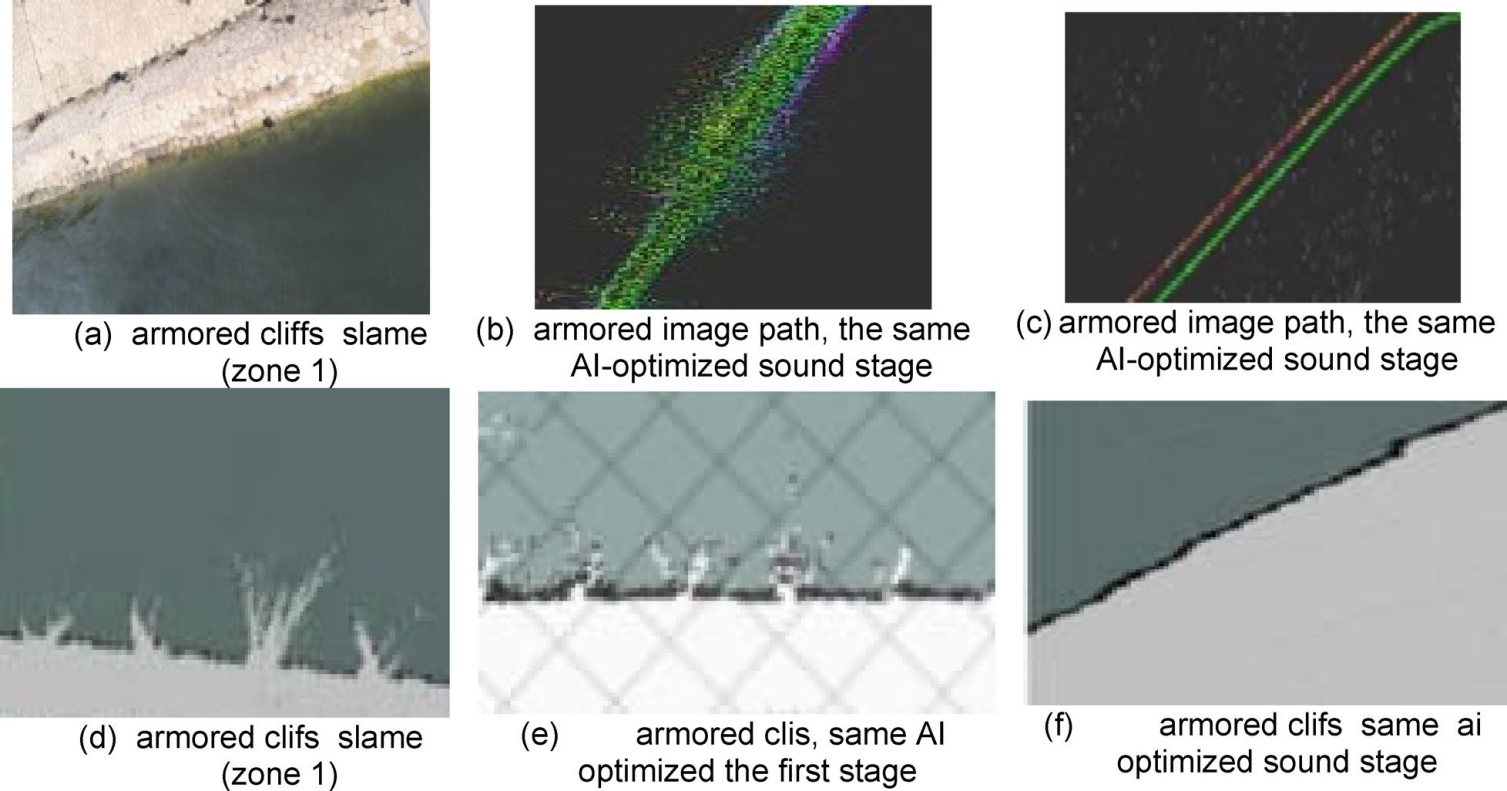
Scanning and optimization results for Zone 1.

The first type is an armoring soft bank in the Tina area, south of Port Said at the 25th-kilometer mark. Its geographical location relative to the navigation channel is shown in Fig. 20. It is an area where the sides of the canal have been stoned because of the aggradation of these layers, which led to the littoral drift of the canal bottoms. Therefore, armoring is considered a datum and represents protection for these cliffs. Therefore, the cliff appears as a straight line as in the cross-section of the area as in Fig. 20. Actual images of the cliff appear in Fig. 16. Part of the data related to the LIDAR sensor scanning appears, which is transmitted from the vehicle to the mainland in the form of distances and angles. It is represented in the SLAM maps using Ross technologies as in Fig. 17 and his optimized. The best navigation path is predicted in the figure. After that, the data is entered into the machine learning optimization programs to predict the missing points, as shown in Fig. 17. Based on this improvement, the path is estimated again as in Fig. 21.

**Fig. 18:**
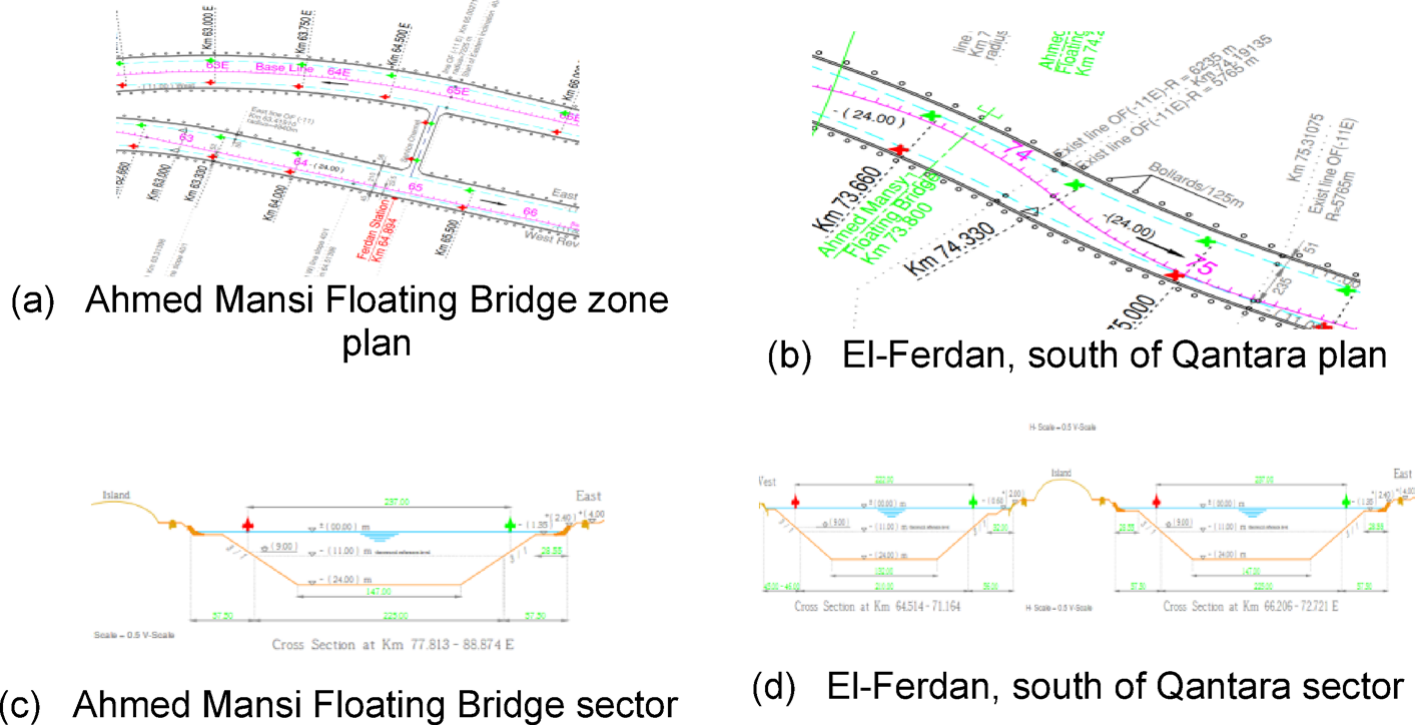
Topographic data for Zone 2.

**Fig. 19:**
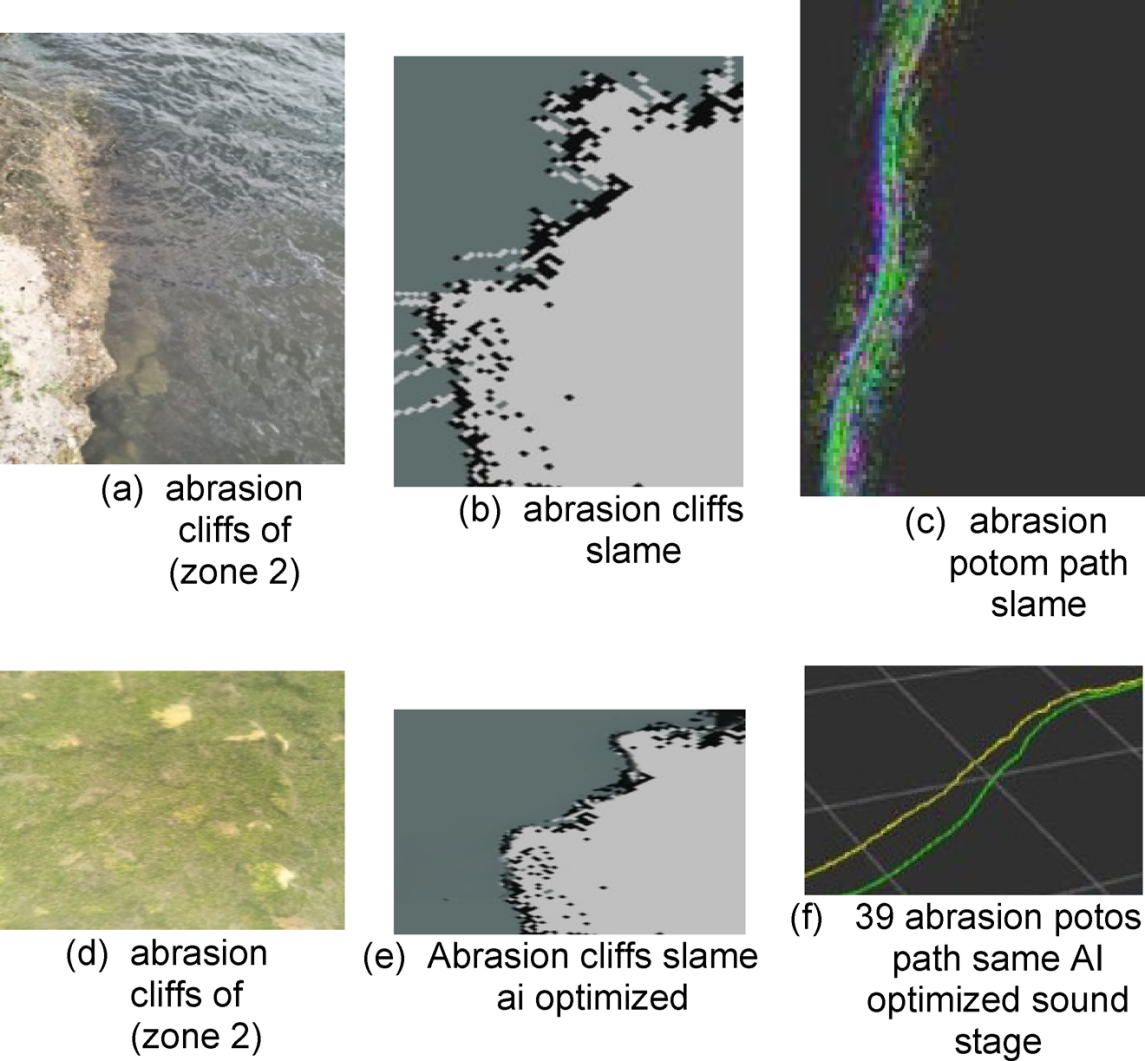
Scanning and optimization results for Zone 2.

The second type is abrasion cliffs in the areas of the Ahmed Mansi Floating Bridge and Al-Fardan, south of Qantara at kilometers 73.6 and 67. Its geographical location relative to the navigation channel is shown in Fig. 18. It is an active coastal zone with rock sediments^23^. Accumulate in it because of shoaled waves resulting from the collision of waves, because of ships passing through this small bay. Therefore, these cliffs are characterized by outcrops because of pore pressure. Also, winds cause the dune process, so some rocky protrusions appear in these cliffs as in the cross-section of the. The real images of the cliff appear in Fig. 19. Part of the data related to the LIDAR sensor scanning appears, which is transmitted from the vehicle to the mainland in the form of distances and angles. It is represented in the SLAM maps using ROS technologies as in Fig. 19, and his optimized. The best navigation path is predicted as shown in the figure. After that, the data is entered into the machine learning optimization programs to predict the missing points. Based on this improvement, the path is estimated again as in Fig. 19.

**Fig. 20:**
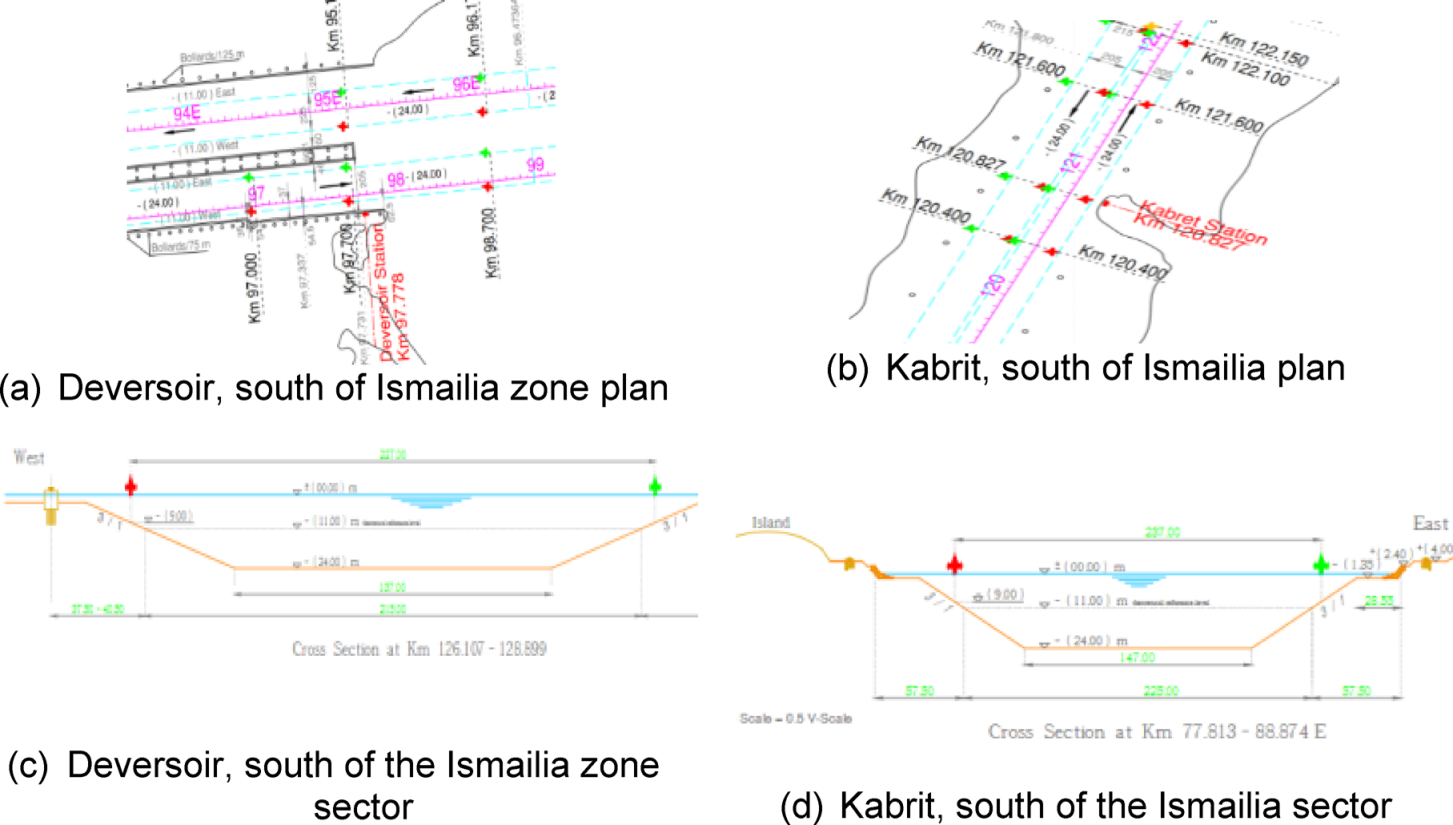
Topographic data for Zone 1.

**Fig. 21:**
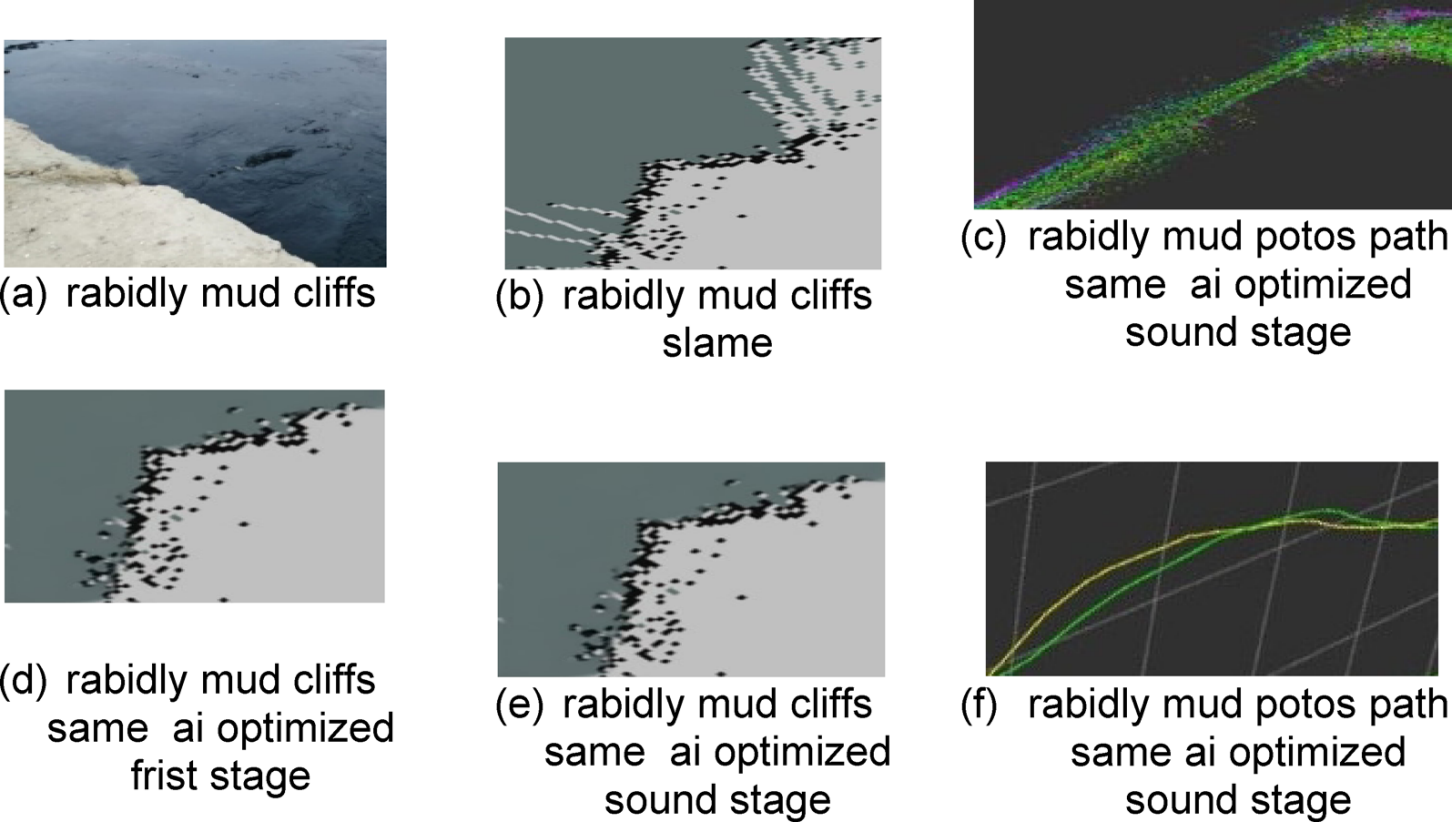
Scanning and optimization results for Zone 3.

The third type is the rabid mud cliffs in the areas of Deversoir and Kabrit, south of Ismailia. At kilometer marks 77.7 and 120.8, its geographical location relative to the navigation channel is shown in Fig. 20 It is an area where silt is abundant and there are many geological factors affecting it, such as the Coriolis current, rip current, and deflation, which leads to Ekman transport and fetch force, which makes the cliffs in some areas fluid mud or liquefaction, like the soil inside the bitter lakes or fore dune, like the cliffs in their northern and southern entrances in Kabrit and Deversoir, as in the cross-section of the area, as in Fig. 20. The real images of the cliff appear as in Fig. 21, and part of the data related to the scanning with the LIDAR sensor appears, which is transmitted from the vehicle to the mainland in the form of distances and angles, and is represented in the SLAM maps using ROS techniques, as in Fig. 21, and his optimized and the best path for navigation is predicted, as in the figure. After that, it enters Data for machine learning optimization programs to predict missing points as shown. Based on this optimization, the path is assumed again as in Fig. 21.

The results are output after the scanning process and organizing the data via the ESP 32 microcontroller and sending the data to the control station on the mainland in the form of distances and angles^24^. as a serial and are output on the Pont operating system organized by the ROS system to simulate the distances and angles to SLAM to display it as a map of the channel erosion and then show a prediction of the path based on the initial scanning data, but the low accuracy of these maps is noted due to the loss of some points as a result of the reflection of the wave emitted from the lidar sensor to a direction different from the lidar receiver as a result of the reflection at a greater angle, which leads to the lack of continuity of the sequence of points about the topographic position^25^. Therefore, the data is entered into the programs working with artificial intelligence, especially machine learning, because the data is serial and the Anaconda Jupiter platform can be used. The points are predicted based on the data of the points adjacent to the missing point from the four directions^23^. The use of the reviewed points may reach 50 points in each direction, depending on the degree of topographic meander of the scanned location^26^.

**Fig. 22:**
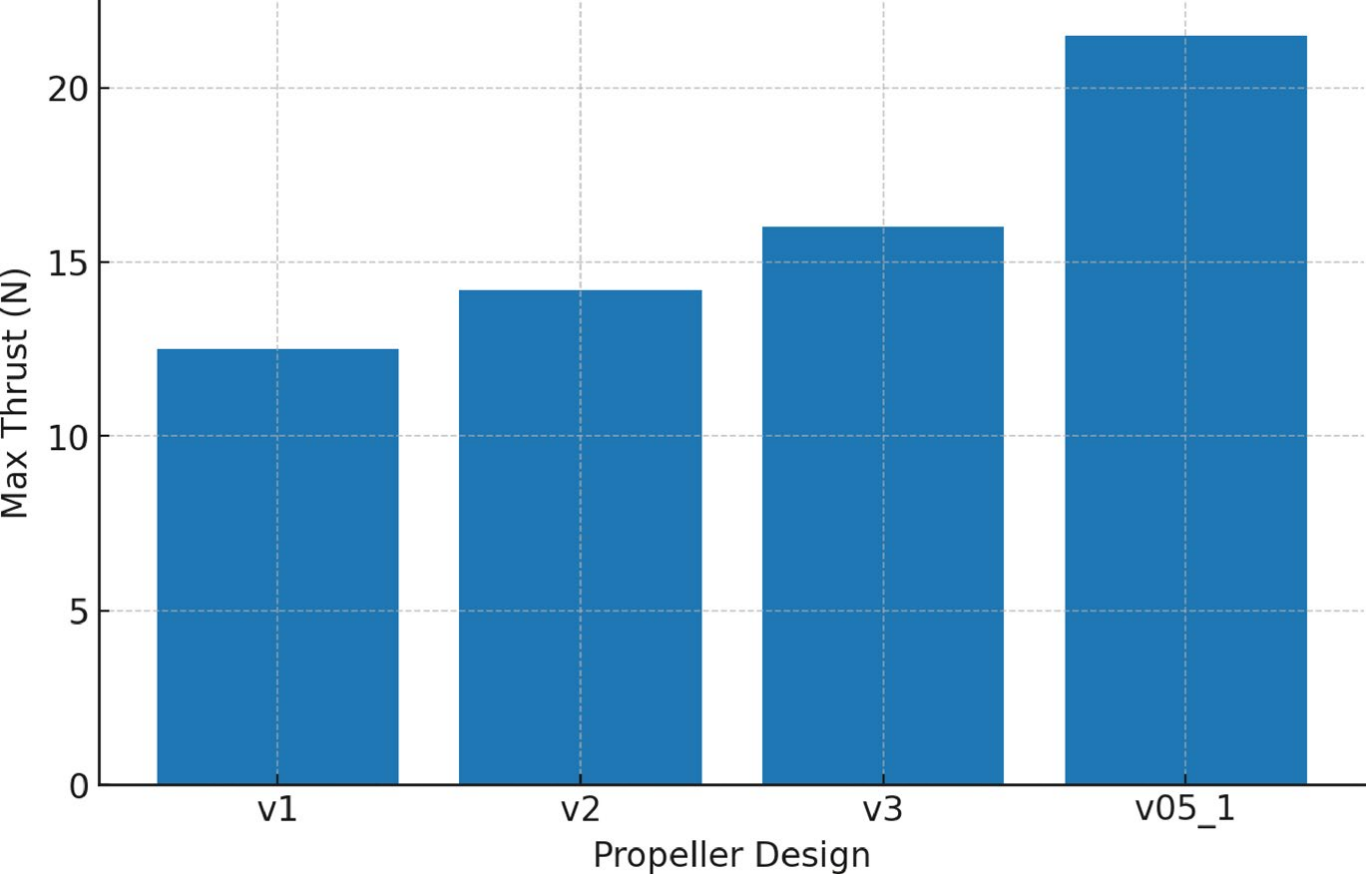
Comparison of propeller designs by max thrust.

Table 5 presents the numerical comparison of maximum and average thrust across the tested propellers, along with observed turbulence levels and efficiency rankings. As shown in Fig. 22, the bar chart provides a clear visual comparison, highlighting the superior performance of the v05_1 design.

These findings justify the selection of v05_1 for the final ROUV prototype, as it provides the optimal balance between thrust, stability, and efficiency. By presenting the thrust data in both tabular and graphical form, the results allow for easier comparison and reinforce the robustness of the design decision.

**Table 5:** Thrust test comparison of propeller designs.

Propeller design	Max thrust (*N*)	Avg. thrust (*N*)	Observed turbulence	Efficiency rank
v1	…	…	High	4
v2	…	…	Medium	3
v3	…	…	Medium–low	2
v05_1	Highest	…	Low	1

To ensure a structured evaluation of the proposed ROUV, a series of tests was conducted, each focusing on a different aspect of system performance. The tests cover stability under controlled conditions, disturbance rejection, SLAM mapping quality, propeller performance, and full-scale navigation trials in the Suez Canal. This structured approach ensures that both subsystem-level and system-level behaviors are validated. A summary is provided in Table 6.

**Table 6:** Summary of performed tests and objectives.

Test ID	Description	Objective
T1	Static stability test in calm water	Validate controller stability and steady-state error
T2	Heading control under flow disturbances	Assess disturbance rejection and trajectory tracking
T3	SLAM performance in a narrow-lane canal section	Evaluate LiDAR + AI mapping continuity
T4	Propeller v05_1 vs. baseline propeller	Measure turbulence effects on scan stability
T5	End-to-end navigation in Suez Canal cliff area	Validate integrated system performance in real-world conditions

The results of these tests demonstrate that the controller achieves robust stability, the redesigned propeller minimizes turbulence, and the AI-based SLAM reconstruction improves mapping continuity in narrow-lane environments.

To position our approach against state-of-the-art methods, we compared the proposed LiDAR + AI ROUV with traditional sonar-based ROUVs^6,12^ and LiDAR-only methods^8,9^. Sonar-based approaches showed high noise and incomplete data in shallow lanes, while LiDAR-only systems produced discontinuous point clouds. In contrast, our integrated LiDAR + AI system improved completeness, stability, and navigation-path accuracy. The comparison is summarized in Table 7.

**Table 7:** Comparison of mapping and navigation performance with state-of-the-art.

Method	Mapping completeness (%)	SLAM continuity (%)	Navigation-path deviation (m)
Sonar-based ROUV^6,9^	68	72	1.8
LiDAR-only ROUV^7,10^	81	84	1.2
Proposed LiDAR + AI ROUV	94	96	0.6

These results highlight that the proposed ROUV achieves higher mapping completeness and continuity, with significantly lower path deviation, thereby offering clear advantages over existing approaches.

## Conclusion

Since the col independently, it would necessarily increase the probability of dangerous invasions in this scenario. Also, the adjusted times for the second vessel are not supported by observation. Recalling heuristic knowledge, after assessing that fairly the two vessels would meet bow to bow, Captain Propush decided the best time to pass port the other vessel between the two oncoming lines would be at 5 min. That is because, as stated above, the col is a reference, used as a threshold, often regarded by sailors to play it safe. Moreover, although COLREGs only become applicable when the vessels are in sight of one another, it is expected for each vessel to detect the presence of any other vessel that presents a risk of collision, and as such, be reflected in the way it plans its passage.

Thus, 3Clifs manoeuvres, whether departing or awaiting a designated time to pass other vessels, always take into account other vessels heeding the first up to a meeting point. At the midpoint gate, field data indicates such an attempt would result in a strategy twice as much as it takes today. Nor the onboard instruments, the monitoring in this site, nor the stakeholders, indicate any drastic course changes at midpoint from a safe navigation standpoint. Additionally, it may be recalled that the Derya Y indirectly influences it.

This study has explored the evolving role of Remotely Operated Underwater Vehicles (ROUVs) in enhancing navigation within narrow maritime lanes, with a particular focus on the Suez Canal. By integrating cutting-edge technologies such as LiDAR, ROS-based SLAM mapping, machine learning, and advanced sensor systems, the proposed 3Clifs ROUV demonstrates how real-time, high-precision underwater surveying can be significantly improved. The research highlights the limitations of traditional sonar-based systems and emphasizes the advantages of adopting intelligent, wireless, and autonomous-capable ROUVs. The ability to continuously monitor and assess topographic and environmental changes in real time can transform the safety and efficiency of navigation in critical trade routes.

Moreover, the historical challenges faced by the Suez Canal further underscore the importance of modernizing guidance systems. The proposed system not only addresses operational limitations but also contributes to a more adaptive, resilient, and data-driven approach to maritime logistics. Future work will focus on extending the autonomy of the ROUV, improving battery efficiency, and conducting broader field trials to validate long-term performance in dynamic underwater environments.

This study demonstrated that integrating LiDAR-based SLAM, AI-driven point-cloud reconstruction, and optimized propulsion design enables more reliable underwater mapping compared to sonar-only and LiDAR-only approaches. Quantitative results (Table 3) showed improved point-cloud completeness, fewer mapping gaps, and reduced navigation-path deviation. The novelty of this work lies in combining sensing, AI optimization, and propulsion improvements into a unified ROUV system, validated in the challenging environment of the Suez Canal. These findings underline the potential of smart ROUVs to enhance navigation safety and efficiency in narrow, shallow maritime lanes.

The novelty of this study lies in combining LiDAR-based SLAM, AI optimization for missing point prediction, and tailored propeller design within a real-world Suez Canal application, which has not been reported in prior works.

## Data Availability

The datasets used and analyzed during the current study are available from the corresponding author upon reasonable request.

## References

[1] McLean, D. L. et al. Enhancing the scientific value of industry remotely operated vehicles (ROVs) in our oceans. *Front. Mar. Sci.* **7**, 220 (2020).

[2] Stanley, D. J., Freeland, G. L. & Sheng, H. Dispersal of mediterranean and Suez Bay sediments in the Suez Canal. *Mar. Geol.* **49** (1–2), 61–79 (1982).

[3] Zulkarnain, O. W., Redhwan, A. A. M., Baba, N. B., Fadhil, M. N. & Rosni, S. Design and development of SelamDrone underwater ROV manoeuvring control. In *Journal of Physics: Conference Series*, 012081 (IOP Publishing, 2021).

[4] Fletcher, M. E. The Suez Canal and world shipping, 1869–1914. *J. Econ. Hist.* **18** (4), 556–573 (1958).

[5] Afifudin, I. N. et al. Optimization design of remotely operated vehicle (ROV) for Madura Strait Area. In *IOP Conference Series: Earth and Environmental Science*, 012029 (IOP Publishing, 2023).

[6] Satria, D. et al. Hydrodynamic analysis of remotely operated vehicle (ROV) observation class using CFD. In *IOP Conference Series: Materials Science and Engineering*, 012014 (IOP Publishing, 2019).

[7] He, Y., Wang, D. B. & Ali, Z. A. A review of different designs and control models of remotely operated underwater vehicle. *Meas. Control*. **53** (9–10), 1561–1570. 10.1177/0020294020952483 (2020).

[8] Dong, M., Chen, D., Ye, N. & Chen, X. Design of ackerman mobile robot system based on ROS and Lidar. In *Journal of Physics: Conference Series*, 012073 (IOP Publishing, 2021).

[9] Holzhüter, H., Bödewadt, J., Bayesteh, S., Aschinger, A. & Blume, H. Technical concepts of automotive lidar sensors: a review. *Opt. Eng.* **62** (3), 031213–031213 (2023).

[10] Christ, R. D. & Wernli, R. L. *The ROV Manual: a User Guide for Remotely Operated Vehicles* (Butterworth-Heinemann, 2013).

[11] Serejo, G. L., dos Santos, V. A., Silva, A. F. B. & Santos, C. G. R. Mobile platform based on ROS and LIDAR for mapping in civil construction. In *International Conference on Computational Science and Its Applications*, 192–205 (Springer, 2023).

[12] Ali, Z. A., Li, X. & Noman, M. Stabilizing the dynamic behavior and position control of a remotely operated underwater vehicle. *Wireless Pers. Commun.* **116** (2), 1293–1309 (2021).

[13] Gerges, M. A. & Stanley, D. J. Assessing hydrography and man’s influence on sediments in the Northern Suez Canal. *Mar. Geol.* **65** (3–4), 325–331 (1985).

[14] Muzammel, C. S., Chakraborty, P., Akram, M. N., Ahammad, K. & Mohibullah, M. Zero-shot learning to detect object instances from unknown image sources. *Int. J. Innov. Technol. Explor. Eng.* **9** (4), 988–991 (2020).

[15] Sultana, M. et al. Object detection using template and HOG feature matching. *Int. J. Adv. Comput. Sci. Appl.* **11** (7), 233–238 (2020).

[16] Faruque, M. A. et al. Ascertaining Polarity of public opinions on Bangladesh cricket using machine learning techniques. *Spatial Inform. Res.* 1–8, (2021).

[17] Leonard, J. J. & Bahr, A. Autonomous underwater vehicle navigation. In *Springer Handbook of Ocean Engineering*, 341–358 (2016).

[18] Altuntas, C. Review of scanning and pixel array-based LiDAR point-cloud measurement techniques to capture 3D shape or motion. *Appl. Sci.* **13** (11), 6488 (2023).

[19] Kellerer-Pirklbauer, A., Avian, M., Kaufmann, V., Niesner, E. & Kühnast, B. Climatic-induced spatio-temporal change of kinematics and ground temperature of rock glaciers and permafrost in the Hohe Tauern Range, Austria. *permafrost—Austrian Permafr. Res. Initiative Final Report*, 17–39 (2014).

[20] Marie, H. S. & Oqda, K. R. Design of a ROUV to guide in narrow lanes. In *International Telecommunications Conference (ITC-Egypt)*, 698–705 (IEEE, 2024).

[21] Li, B., Ma, Z. & Zhao, Y. 2D Mapping of mobile robot based on micro-ROS. In *Journal of Physics: Conference Series*, 012030 (IOP Publishing, 2022).

[22] Tsuruoka, S. et al. ROS evaluation for a series of CNTs and their derivatives using an ESR method with DMPO. In *Journal of Physics: Conference Series*, 012029 (IOP Publishing, 2013). 10.1088/1742-6596/429/1/012029 PMC4544674 26300949

[23] Bingham, A., Hadoux, X. & Kumar, D. K. Implementation of a safety system using ir and ultrasonic devices for mobility scooter obstacle collision avoidance. In *5th ISSNIP-IEEE Biosignals and Biorobotics Conference (2014): Biosignals and Robotics for Better and Safer Living (BRC)*, 1–5 (IEEE, 2014).

[24] Chikhalikar, A., Ravankar, A. A., Luces, J. V. S., Tafrishi, S. A. & Hirata, Y. An object-oriented navigation strategy for service robots leveraging semantic information. In *2023 IEEE/SICE International Symposium on System Integration (SII)*, 1–6 (IEEE, 2023).

[25] DIY Submersible ROV : 8 Steps (with Pictures) - Instructables. https://www.instructables.com/DIY (Accessed 14 Apr 2024).

[26] Liu, M., Chen, M., Wu, Z., Zhong, B. & Deng, W. Implementation of intelligent indoor service robot based on ROS and deep learning. *Machines* **12** (4), 256 (2024).

